# Stuck on a Plateau? A Model-Based Approach to Fundamental Issues in Visual Temporal-Order Judgments

**DOI:** 10.3390/vision2030029

**Published:** 2018-07-16

**Authors:** Jan Tünnermann, Ingrid Scharlau

**Affiliations:** Psychology, Paderborn University, 33098 Paderborn, Germany

**Keywords:** temporal-order judgments, modeling, theory of visual attention, TVA, range of indecision, encoding reset

## Abstract

Humans are incapable of judging the temporal order of visual events at brief temporal separations with perfect accuracy. Their performance—which is of much interest in visual cognition and attention research—can be measured with the temporal-order judgment (TOJ) task, which typically produces S-shaped psychometric functions. Occasionally, researchers reported plateaus within these functions, and some theories predict such deviation from the basic S shape. However, the centers of the psychometric functions result from the weakest performance at the most difficult presentations and therefore fluctuate strongly, leaving the existence and exact shapes of plateaus unclear. This study set out to investigate whether plateaus disappear if the data accuracy is enhanced, or if we are “stuck on a plateau”, or rather with it. For this purpose, highly accurate data were assessed by model-based analysis. The existence of plateaus is confidently confirmed and two plausible mechanisms derived from very different models are presented. Neither model, however, performs well in the presence of a strong attention manipulation, and model comparison remains unclear on the question of which of the models describes the data best. Nevertheless, the present study includes the highest accuracy in visual TOJ data and the most explicit models of plateaus in TOJ studied so far.

## 1. Introduction

Psychometric distributions of temporal-order judgments are assumed to be monotonic and typically yield S-shaped functions [1,2], which are, in accordance with psychophysical tradition, often interpreted as cumulative normal distributions or logistic functions, a function type which is common in psychophysics [3,4]. Such functions are usually characterized by two descriptive parameters, the so-called point of subjective simultaneity (the location of its center on the *x*-axis), and a measure of the precision of discrimination (its slope). Much theorizing in psychology is built upon these parameters.

Interestingly, some researchers also hinted at distributions that show a more or less pronounced plateau in their center, around the point of subjective simultaneity. At such a plateau, the judgment frequency does not change depending on the temporal interval so that the psychometric function is flat in its range. Models that assume a range of indecision with a strong influence on temporal judgments predict such plateaus. Such a range of indecision can result, for instance, from a threshold in temporal-order perception below which no order can be discriminated or from a process of switching between channels which needs time (for an early discussion see [1]). Sometimes, but very rarely, plateaus are indeed observed and discussed [5], and a variety of studies indicate such data patterns without discussing them e.g., [6,7,8,9,10,11,12]; the study by Sternberg, Knoll and Gates [11] gives some apparently impressive examples on the level of individual participants. That they are so rarely visible or reported may be due to the fact that temporal intervals are often too broadly spaced to let plateaus show up and that summary data are reported. Furthermore, data are usually not precise enough in terms of repetitions of conditions.

Until now, however, it is not clear that there are indeed plateaus in TOJs. Even though data patterns sometimes show them, their existence is disputable: variability in visual TOJ data, which is especially large in the critical central range, leaves open the possibility that they result from chance alone. For establishing their presence or absence, two conditions have to be met: (1) precise data, in which noise does not obscure the measurements at the most important points; and (2) narrowly spaced temporal intervals that guarantee that the plateau can spread out across several intervals. The present study meets these conditions in the case of visual TOJs.

So far, mainly one cause of these plateaus has been discussed in the literature, namely a decision-level process. In this view, the plateau originates from superposing a non-deterministic decision function on the arrival time difference function [1,13]. The arrival time difference function resembles the aforementioned S-shaped functions of “probe first” reports over the stimulus onset asynchronies (SOAs). The “probe” is one of the two targets, the other is the “reference”; the meaning of these labels will be discussed later. The order arrival function is combined with a decision function, mapping the arrival time difference to the judgments (cf. [1]). A perfect deterministic observer maps all negative arrival time differences to the “probe first” judgments and all positive ones to “probe second”. However, modern models use probabilistic stimulus encoding models and sometimes impose a minimal temporal difference constraint on the decision mechanism [13,14,15]. These process-based models allow for explicit modeling of the processes that lead to TOJs. From a scientific point of view, this is much more useful than the descriptive parameters of traditional psychometric functions. As their name indicates, such descriptive parameters simply describe the psychometric function of the judgments, with little or no relationship to potential processing that causes the distribution of judgments.

According to Alcalá-Quintana and García-Pérez [13], plateaus indicate ranges of indecision (as already argued by Neumann and Scharlau [5]). This is a range of arrival time differences in which observers are unable to determine the temporal order. Hence, in this view, the plateau is caused by a mechanism that occurs *after* the stimuli are encoded.

In an alternative model considered in the present paper, the plateau is caused by a processing reset elicited by a second stimulus quickly appearing after a first one. Its cause thus lies *before* or at the beginning of stimulus encoding, leaving the decision mechanism perfectly deterministic. This model is an extension of our earlier work [15,16,17,18,19,20,21], in which we derived a TOJ model from Bundesen’s [22,23] theory of visual attention (TVA). Notably, TVA has been successfully applied in many domains of attention research and different experimental paradigms (e.g., whole report, partial report, and attentional dwell time experiments). The results for TOJs so far are promising. Parameter estimates are well within the ranges of other TVA paradigms (cf. Tünnermann [18], pp. 153–154). Therefore, it is worthwhile to further refine the TVA-based TOJ model in order to capture possible plateaus.

Within the TVA-based TOJ model, perceived order depends directly on stimulus entry into visual short-term memory, leading to a strictly monotonic function. The model thus faces the challenge to account for the plateau. Of course, a new parameter could always be added to a model to capture some assumed process or pattern in the data. This is not the approach we take. As will be discussed below in Section 2.3, we take an already existing parameter of TVA which formerly canceled out in the TVA-based TOJ model, and tie it to an additional mechanism which results in a plateau, thus keeping the model sparse.

The basic idea of the proposed mechanism is that if one stimulus is presented in close temporal succession to the other, processing is reset for both stimuli. That is, although encoding started when the first stimulus was presented, the onset of the second stimulus causes a withdrawal of resources (a reset). Thereafter, resources are distributed to both stimuli. This leads to an SOA range for which it is likely that both stimuli arrive at virtually the same time in TVA’s visual short-term memory, an SOA range during which encoding order is random, causing a plateau.

Note that TVA is a fairly general model of visual selection and recognition. If the new model can successfully handle the plateaus (supposed they exist), this would extend and refine the TVA-based approach and be an important step toward a unified model in attention research. In comparison, Alcalá-Quintana and García-Pérez’ approach is rather specific.

The focus on models in the present paper serves a further purpose. Modeling is often suggested as a means to advance scientific progress in psychology. Although it may seem so at first sight, the application or construction of models is very often not straightforward. Compared to areas of science in which basic concepts are well known and mathematically defined or observational data are not very noisy, psychology faces a variety of problems that are hard to tackle. Despite the many advantages of modeling (as discussed, for instance, by Taagepera [24]), it is not yet entirely clear how progress can be made with the help of models. For instance, we have few experiences if different quantitative criteria for evaluating the appropriateness of models do not agree, or in deciding between models when data fit and theoretical considerations do not coincide. Is it, for instance, advisable to add a specific, but interpretable parameter to a general model if it augments its data fit for a specific experimental paradigm, but is of no use in other situations that the model is meant to explain? These are scientific problems that psychology still has to explore, and the present paper wants to contribute to this exploration.

In short, we show in the present paper (1) that central plateaus are present in even in the most simple binary visual TOJs if sufficiently accurate data is collected; (2) that they are well described by a process-based model that assumes a range at which participants cannot resolve the order and guess the decision; (3) and that a novel model based on Bundesen’s TVA is able to describe the data without calling for decision-level indeterminism. We will (4) pay close attention to the many intricate problems of the connections between models and data.

## 2. Material and Methods

### 2.1. Exponential Race Models of Temporal-Order Judgments

Both TOJ models that are considered in the present paper are based on an exponential race model. That is, they model the encoding of the targets probabilistically using exponential distributions. These distributions are found in some natural processes and have also been used to model stimulus encoding. In García-Pérez and Alcalá-Quintana’s model [13,25], this choice is motivated by the exponential distribution’s “crucial property that the time at which sensory signals reach a central mechanism cannot precede the onset of the stimulus triggering that signal” ([13], pp. 973), a logical constraint to which other models (e.g., normally distributed arrival times [14]) do not adhere. In our TVA-based TOJ model, the exponential race is a fundamental property inherited from TVA. In TVA, the exponential distribution enjoys strong empirical support in that complex experimental data can be closely fitted [26]. Because the exponential race encoding is shared by both considered models, a unified mathematical description of this model part is provided in the following.

The probability $P_{p^{1st}}$ that the probe stimulus *p* is encoded before the reference stimulus *r* can be described as

(1) $$P_{p^{1st}}\left(v_{p},v_{r},SOA\right)=\left\{\begin{array}{cc} 1-e^{-v_{p}\left|SOA\right|}+e^{-v_{p}\left|SOA\right|}\left(\frac{v_{p}}{v_{p}+v_{r}}\right),\; & \mathrm{for}\;SOA<0,\\ e^{-v_{r}\left|SOA\right|}\left(\frac{v_{p}}{v_{p}+v_{r}}\right),\; & \mathrm{for}\;SOA\geq 0, \end{array}\right.$$

In an exponential race model, a stimulus *x* with rate $v_{x}$ is encoded until time *t* with a probability of $1-e^{-v_{x}t}$. On this basis, the equations above can be interpreted as follows: At negative SOAs (where the probe is shown before the reference), the probability that the probe is encoded first consists of two additive components: The first is $1-e^{-v_{p}\left|SOA\right|}$, the probability that stimulus *p*, processed at rate $v_{p}$, is encoded before the reference stimulus *r* is presented. If this did not happen (which has a probability of $e^{-v_{p}\left|SOA\right|}$), *p* is encoded first with the probability $v_{p}/\left(v_{p}+v_{r}\right)$, according to Luce’s choice axiom [27].

García-Pérez and Alcalá-Quintana derive their model differently, starting with a bilateral exponential distribution of the arrival time difference (which is the probability distribution of the difference of the arrival times of two exponentially distributed processes [25]). Despite the different description, the encoding models are mathematically identical. García-Pérez and Alcalá-Quintana’s rate parameters $\lambda_{t}$ and $\lambda_{r}$ correspond to TVA’s $v_{p}$ and $v_{r}$ in the description above (for consistency, we use the subscripts “*p*” and “*r*” below). Note that this is true only if García-Pérez and Alcalá-Quintana’s bias parameters are set to neutral values and the range of indecision is set to zero, which reflects a deterministic decision. In other words, the basic encoding model is identical in both approaches. Differences arise from the additional mechanisms and parameters, which are described in the following.

### 2.2. Cause of a Plateau: Range of Indecision

In García-Pérez and Alcalá-Quintana’s model, the plateau arises because participants need a minimum temporal separation between the signals to determine the order. Otherwise, they perceive the stimuli as simultaneous (or are highly uncertain about the order [28]). Forced to judge, they thus respond at a chance level. This results in a range of indecision, and therefore the plateau. The plateau’s location on the SOA axis is determined by an additional parameter τ which models a net delay that may occur because of latencies in the processing of each stimulus. The plateau can also be shifted vertically (on the “proportion correct” axis) via a response bias parameter ξ.

The psychometric function of reporting a stimulus *p* as appearing first is then given as

(2) $$\begin{array}{ccc} P_{p^{1st}}^{\mathrm{range}}\left(\lambda_{p},\lambda_{r},\delta ,\tau ,\xi ,SOA\right) & = & P_{p^{1st}}\left(\lambda_{p},\lambda_{r},-\delta +SOA+\tau \right)\\ & + & \left(1-\xi \right)(1-P_{p^{1st}}\left(\lambda_{p},\lambda_{r},\delta +SOA+\tau \right)\\ & & \;-\left(1-P_{p^{1st}}\left(\lambda_{p},\lambda_{r},-\delta +SOA+\tau \right)\right))\\ & + & 1-P_{p^{1st}}\left(\lambda_{p},\lambda_{r},\delta +SOA+\tau \right). \end{array}$$

The first term models “probe first” percepts. The second term represents “simultaneous” percepts, and the third term captures “reference first” percepts. A different path that leads to this model is described in [13]. The equation provided above was constructed using the probabilities for “probe first” percepts as stated in Equation (1).

Importantly, and in contrast to the *encoding reset model* presented below, the plateaus in this approach originate from a time interval or an area (−δ to δ) during which the order cannot be discriminated. Because this is the crucial feature for the present study, the term *indecision range model* will be used henceforth to refer to the model.

### 2.3. Cause of a Plateau: Encoding Reset

The *encoding reset model* is an alternative explanation for central plateaus ([18], p. 78). It combines Bundesen’s TVA [22] with the assumption that, under certain conditions, the processing race is abolished and started anew. At its core is the $t_{0}$ parameter, which belongs to TVA’s basic parameters. It represents the maximum ineffective exposure duration. That is, stimuli presented for shorter intervals are not processed at all [22]. In TOJs, the parameter usually cancels out because the $t_{0}$ values of both stimuli are typically identical. If the encoding races of both stimuli are delayed by the same $t_{0}$ value, there is no effect on the judgments. However, in the *encoding reset model* tested in the present study, $t_{0}$ has an important consequence: The main idea of the model is that, if the second stimulus appears within the interval $t_{0}$ (typically 10 to 20 ms) of the first stimulus, all processing is halted and after a “reset” both stimuli race again with a common start.

To obtain a smooth psychometric function, $t_{0}$ is modeled as a normally distributed random variable with a variance of ${s_{0}}^{2}$. A normal distribution for $t_{0}$ was also suggested by Dyrholm et al. [29] to account for trial-by-trial variability typically observed for $t_{0}$:

(3) $$\begin{array}{c} P_{p^{1st}}^{\mathrm{reset}}\left(v_{p},v_{r},t_{0},s_{0},\tau ,SOA\right)=\left\{\begin{array}{c} \begin{array}{cc} \Phi \left(\frac{SOA+t_{0}+\tau }{s_{0}}\right)\cdot \frac{v_{p}}{v_{p}+v_{r}} & \\ +\left(1-\Phi \left(\frac{SOA+t_{0}+\tau }{s_{0}}\right)\right)\cdot P_{p^{1st}}\left(v_{p},v_{r},SOA+\tau \right), & \\ & \mathrm{if}\;SOA+\tau \leq 0, \end{array}\\ \begin{array}{cc} \left(1-\Phi \left(\frac{SOA-t_{0}+\tau }{s_{0}}\right)\right)\cdot \frac{v_{p}}{v_{p}+v_{r}} & \\ +\Phi \left(\frac{SOA-t_{0}+\tau }{s_{0}}\right)\cdot P_{p^{1st}}\left(v_{p},v_{r},SOA+\tau \right), & \\ & \mathrm{if}\;SOA+\tau >0, \end{array} \end{array}\right. \end{array}$$

where Φ(x) is the cumulative distribution function of the standard normal distribution and $P_{p^{1st}}\left(v_{p},v_{r},SOA\right)$ the function of a “probe first” percept as defined in Equation (1).

**Figure 1:**
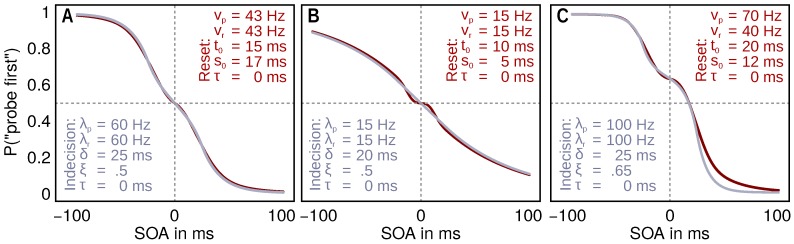
(**A**) for many cases, the two models predict similar “probe first” curves, though assuming different values for the rate parameters; (**B**) the *encoding reset model* can produce shallow overall curves with a crisp plateau. For the *indecision range model*, the dispersion of the plateau is always similar to that of the curve in general, producing no noticeable plateaus for shallow curves like this one; (**C**) the vertical plateau position can shift independently of the processing rate in the *indecision range model*. In this example, both rates are equal ($\lambda_{p}=\lambda_{r}$). By contrast, in the *encoding reset model*, a similar vertically shifted plateau can only be achieved by a difference in the processing rates ($v_{p}>v_{r}$ shifts the plateau upwards as in this example). Note that the weaker reference rate results in a more shallow curve at the positive stimulus onset asynchronies (SOA). An interactive demonstration can be found at [30].

In this model, the plateau does not originate from a temporal range of indecision. Instead, the appearance of stimulus in the visual field within the $t_{0}$ period of an earlier stimulus resets the system and both stimuli start again simultaneously. The order decision in this model is entirely deterministic, based on the visual short-term memory arrival times. Thus, the encoding-reset model draws on a quite different general idea to explain plateaus in TOJ data. An encoding reset is a mechanism which might play a role in very different situations with a quick succession of stimuli, such as the attentional blink. (One might wonder whether the *encoding reset model* is indistinguishable from a model which includes the perception of simultaneity that would be triggered whenever a stimulus pair falls within the reset interval. This, however, is not the case. In the *encoding reset model*, despite the reset, stimuli can still be perceived as not simultaneous when they are processed with unequal rates. Unequal processing rates shift the plateau vertically away from 0.5, which is the expected position for perceiving simultaneity. Even if a subsequent decision bias that also shifts the plateau is incorporated (as in the *indecision range model*), the interactions of the parameters and the exact plateau curves show differences (see Figure 1C).)

Note that both plateau models can produce relatively similar psychometric curves. However, these result from different interactions between the parameters, and there are conditions (e.g., when there are vertically displaced plateaus in the data) in which the predictions are clearly different. This is illustrated in Figure 1. In addition, an interactive demonstration, in which the curves can be compared for arbitrary parameter choices, can be found at [30].

### 2.4. General Experimental Procedure

**Figure 2:**
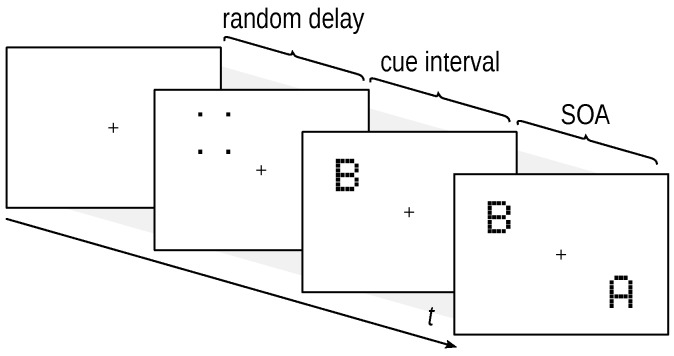
Exemplary display sequence of a cued temporal-order judgment trial. The SOA, the stimulus onset asynchrony, is the delay between the two targets. The trial begins with a random delay which is, in Experiment 2, followed by a cue which leads the cued target by a fixed cue interval. Stimuli are not drawn to scale.

To compare the plateau-generating models, we recorded data from simple TOJs, as illustrated in Figure 2, in which participants report which of two stimuli appeared first. Experiment 1 tested the basic TOJ. Experiment 2 used a cued TOJ, in which attention is biased towards one of the stimuli.

#### 2.4.1. Stimuli

Stimuli were 22 uppercase letters from the Latin alphabet excluding those that are easily confusable (I, Q, V, and Y). The letter stimuli were made of little squares on a 5 × 7 grid that extended 0.8×1.3° of visual angle. Targets of this font have been used in several previous experiments [16,17]. Examples are presented in Figure 2. Letters were displayed in black on a light gray background.

#### 2.4.2. Procedure

The experiment was conducted in sound-proof booths. The participants observed the 21${}^{\prime \prime }$ monitor from a distance of 59 cm, their chin fixed by a chin rest.

A trial began with the presentation of a fixation mark (a small cross) for a randomly chosen value of 500 to 1000 ms in the center of the monitor. After the presentation of a fixation mark, two letters were shown at two random of four possible positions around fixation ([$4^{\circ },4^{\circ }$], [$-4^{\circ },4^{\circ }$], [$-4^{\circ },-4^{\circ }$], [$4^{\circ },-4^{\circ }$] visual angle).

The stimuli were temporally separated by the SOA (stimulus onset asynchrony), which varied from −100 to 100 ms. At negative SOAs, the probe stimulus was shown first. Subsequently, the reference stimulus was presented. At positive SOAs, the reference stimulus led and was followed by the probe. In cued TOJs, the probe is the one preceded by the cue per definition. Hence, Figure 2 depicts a cued TOJ trial with a negative SOA. In neutral conditions, the probe–reference assignment is arbitrary. Note that we also included an SOA of zero, at which both stimuli were shown simultaneously.

After the two letters had appeared, the participants indicated their order by pressing the respective letters on the keyboard. The participants were instructed to be as accurate as possible. They could report the order by entering letters in the perceived order, or enter them in any order and toggle the report which was presented at fixation afterward. If the targets were perceived as simultaneous, the participants were instructed to guess. The “Enter” key started the next trial.

Importantly, to produce highly accurate data to probe the plateau-generating mechanisms, a large number of narrowly spaced SOAs and a large number of repetitions is necessary. In particular, 21 SOAs from −100 to 100 ms at intervals of 10 ms were used. Each SOA was repeated 240 times in an intermixed manner in Experiment 1 (Experiment 2 had an uneven trial distribution, which will be explained later). This means that, in each experiment, up to 5040 trials were presented, which is about ten times as much as in usual visual TOJ experiments. Participants appeared for up to 20 blocks, typically performing two or three in one session.

### 2.5. Experiment 1: Basic TOJs

The experiment assessed visual TOJs according to the general procedure described above (Section 2.4). Because both stimuli were equally relevant and many very small SOAs with many repetitions were used, we expect a symmetric psychometric function with a central plateau.

### 2.6. Participants

**Figure 3:**
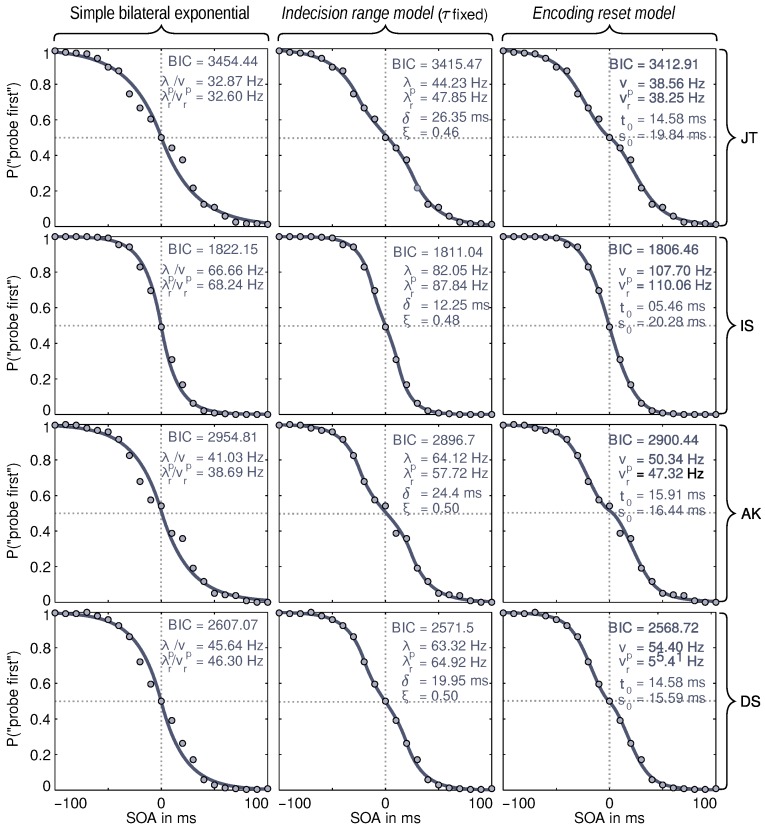
Data, fits, and parameters in Experiment 1. The rows show the different participants, labeled with initials. The columns are the different models, identified in the heading at the top. Data points are the frequencies of the judgment “probe first” for the 21 stimulus onset asynchronies (SOAs) studied. In each panel, the respective Bayesian information criterion (BIC) score and all used parameters are given: parameters λ and *v* are the speed parameters for probe and reference (identified by the subscripts *p* and *r*). The δ parameter is the size of the range of indecision and bias ξ its location on the *y*-axis. Threshold $t_{0}$ is the minimal effective exposure duration and $s_{0}$ its standard deviation. For detailed definitions of the parameters, please refer to Section 2.

The experiment had four participants, including the authors. All of them were informed about the purpose of the experiment. They appeared for ten sessions distributed over several days so that each produced very precise data. The authors are identified by their initials in Figure 3.

### 2.7. Stimuli and Procedure

Letter targets were presented as described in the General Procedure section (Section 2.4).

### 2.8. Results

The data was fitted on the subject level with a modified (to account for the new models) version of Alcalá-Quintana and García-Pérez’s [13] framework. The evaluation included the TVA-based *encoding reset model* and the *indecision range model*. The *indecision range model* was used without error parameters. Additionally, the τ parameter (which is responsible for the plateau position on the *x*-axis) was fixed at zero. Omission of these two parameters ensured that both models have the same complexity with four parameters (see second and third column of Figure 3). These parameters were not relevant because the τ parameter is not expected to vary in a simple visual TOJ, and the error parameter is not necessary because the participants were highly trained during the experiment. A third model based on a simple bilateral exponential distribution of the arrival times was included to provide a comparison with a model without a plateau-generating mechanism. This simple model is a special case of both the TVA-based *encoding reset model* and the *indecision range model*.

The models are compared with the help of the Bayesian information criterion (BIC), a common model comparison score. The BIC score is a relative measure: in a comparison, the model with the smaller score is to be preferred over the model with the larger score. The BIC takes the quality of the fit and the complexity of the model into account [31]. Taking the complexity into account was necessary for the comparison with the simple model because, in contrast to the two other models, it had a different number of free parameters.

The model in the left column of Figure 3 shows the strongest deviations from the data. This is the model without any mechanism, which generates a central distortion. The visual impression is reflected in the BIC scores, which indicate strong evidence against this model (differences of 10 and larger are interpreted as strong evidence). Hence, it can be concluded that some mechanism that generates such plateaus needs to be included to model TOJ data accurately.

The model in the second column is the *indecision range model* with τ fixed at zero. In this model, the plateau is controlled by the resolution parameter δ, which is estimated at values between 12 and 26 ms. The vertical position of the plateau is controlled by ξ, the potential response bias. Here, it was estimated at values around 0.5, which indicates the absence of bias. In the simple visual TOJ of Experiment 1, no bias is expected. Still, the parameter was allowed to vary in order to provide the model with the same number of degrees of freedom for their plateau mechanisms as the TVA-based TOJ model.

In the right column, the results of the TVA-based TOJ model, the *encoding reset model*, are shown. Here, $t_{0}$ and its standard deviation $s_{0}$ (due to trial-by–trial variability, cf. [29]) control the plateau via the reset mechanism. The $t_{0}$ parameter is estimated at values of approximately 15 ms with a similarly sized standard deviation.

Substantial models have to yield plausible parameter values, which we will discuss first, before turning again to the BICs to compare the two models with plateau-generating mechanisms. The estimates are shown together with the data and fitted curves in Figure 3.

For the processing rates of the two stimuli, we have to ask whether they are in a plausible range. This is indeed the case. Values of 40 to 80 Hz reflect relatively strong performance that is plausible because the participants were well-trained. TVA estimates from simple visual tasks commonly range from 30 to 50 Hz (cf. Table 2 in [18], p. 153; Note: $v_{p}+v_{r}=C$). For the TOJ, in earlier studies, we also often found similar rates (cf. Table 3 in [18], p. 154). Participant IS is a little, but not critically, above this range.

Interestingly, the pattern of processing speeds (*v* and λ) differs a bit between the two models: For three participants, the *encoding reset model’s* estimates are about 10 Hz lower; for IS, the difference is the other way around and double in size. This difference is interesting because both *v* and λ are processing speed parameters and thus should result in a similar pattern. A difference of 10 Hz is neither critically large nor negligible. (In the passing, it may be noted that the model without plateau-generating mechanism yields much smaller rates. This is because it has to lower the rates in order to approximate the more shallow slope in the center of the psychometric distributions.)

The other parameters differ in meaning between the two models and will thus be discussed without comparison. The parameter δ in the *indecision range model* corresponds to the range of indecision. Its values range between 12 and 26 ms. Looking at the interval of uncertainty in unimodal visual synchrony judgments, these are plausible estimates. Several studies [10,28,32] indicate summary values of roughly 30 ms for visual TOJs. Note, however, that there is no canonical interpretation of the interval of uncertainty so that these values cannot directly be compared with δ. The plateaus are usually at 0.5 on the *y*-axis, that is, there is no bias for one of the stimuli (ξ=0.5). Note that the stimuli were absolutely indistinguishable for the participants so that no bias in favor of one or the other is possible. Thus, the very small deviations from ξ=0.5 for JT and IS as well as any λ differences within participants reflect uncertainty in the data.

In the *encoding reset model*, the thresholds $t_{0}$ are around 15 ms for three participants and around 5 ms for the fourth. These are again plausible values (cf. Table 2 in [18], p. 153). The variance parameter $s_{0}$ yields consistent estimates of 16 to 20 ms.

Overall, both models with plateau-generating mechanisms yield good fits. These 4-parameter versions of both models closely approximate the judgment distributions. A possible, but not clear, exception is participant AK whose data indicate more than a simple plateau in the center of the distribution. Whether this is a consistent pattern or is caused by variability is difficult to decide.

For three of the four participants (JT, IS, DS), the lower BIC score signifies that the TVA-based *encoding reset model* is to be preferred. For participant AK, the BIC is in favor of the *indecision range model*. Taking the BIC values into account, differences between model fits exist but are small. All in all, therefore, both models with plateau-generating mechanisms capture the data adequately and explain them with interpretable, process-based parameters. For deciding between the models, more information is needed.

## 3. Experiment 2: TOJs with Exogenous Attention Manipulation

Experiment 2 further investigates the question whether an early pre-race reset and a deterministic decision rule can explain the distortions of TOJ curves typically attributed to a non-deterministic decision mechanism. To this end, an attention condition was included: if attention is shifted toward one of the targets, “prior entry” arises, the earlier perception of the attended stimulus compared to the unattended one, caused by a relative effect on the stimulus processing latencies [33]. Thus, a skewed psychometric function can be expected. The behavior of the central plateau under such conditions may provide cues for deciding between models and their explanations. In order to make sure that the attentional manipulation is strong enough, we used a peripheral cue. Such cues have a strong influence on the psychometric distribution [9,32]. This variability offers the opportunity to be less or more well described by the interaction of the model parameters observed in Experiment 1. For instance, in the *encoding reset model*, a vertical displacement of the plateau can only come along with a difference in processing rates that causes deviations from symmetry at the outer parts of the curve. In the *indecision range model*, a vertical plateau displacement can occur via the bias parameter ξ even at equal processing rates (see Figure 1).

### 3.1. Participants

Four participants produced data in five to ten sessions.

### 3.2. Stimuli

Stimuli were the same as in Experiment 1 and as described in Section 2.4. In addition to the targets, a peripheral cue consisting of four small squares was presented before one of the targets (the probe target). The squares were adjacent to (but not touching) the outermost possible squares of the target letters in the upper and lower right and left corners. The cue was only weakly visible under these presentation conditions.

### 3.3. Procedure

The procedure was the same as in the previous experiment and as outlined in Section 2.4, except that the peripheral cue was presented in a random half of the trials. The cue was presented with an onset interval of 140 ms either before the probe stimulus (which can be the first or before the second target, depending on the sign of the SOA).

Because this experiment included two intermixed conditions, neutral and cue, the overall amount of trials was split up between the two. Moreover, most participants were not available for as many sessions as in Experiment 1. To counter the loss of trials that go into each psychometric function, uneven numbers of repetitions and shifted SOAs (in the cue condition) were used. These adjustment aimed at concentrating repetitions at critical central SOAs where the curves are most informative. At the extreme SOAs, participants are typically always correct, and hence fewer trials suffice. In particular, the repetition distribution (8, 10, 12, 14, 16, 18, 20, 22, 24, 26, 28, 26, 24, 22, 18, 16, 14, 12, 8) was used in this experiment, whereas, in Experiment 1, all SOAs were repeated 24 times. The SOAs in the neutral trials were as in Experiment 1 and that of the attention condition ranged from −40 to 160 ms in intervals of 10 ms, shifting it roughly in the expected direction. Such adjustments to repetition counts and SOA locations are not uncommon (e.g., see [11,16]). In hindsight, and as will become clear in the discussion of the results, these adjustments and the lower overall trial count may be problematic.

### 3.4. Results

The model without any plateau-generating mechanism was excluded because it yielded an insufficient fit in Experiment 1. The other two models were again used, and the delay parameter τ was added to both. This parameter is part of the Alcalá-Quintana and García-Pérez’ original model and, as mentioned above, determines the location of the plateau on the *x*-axis. It has the same effect in the TVA-based TOJ model, although the interpretation that this is a simple horizontal shift is a simplification of the complex processes associated with a peripheral cue (cf. [17]).

**Figure 4:**
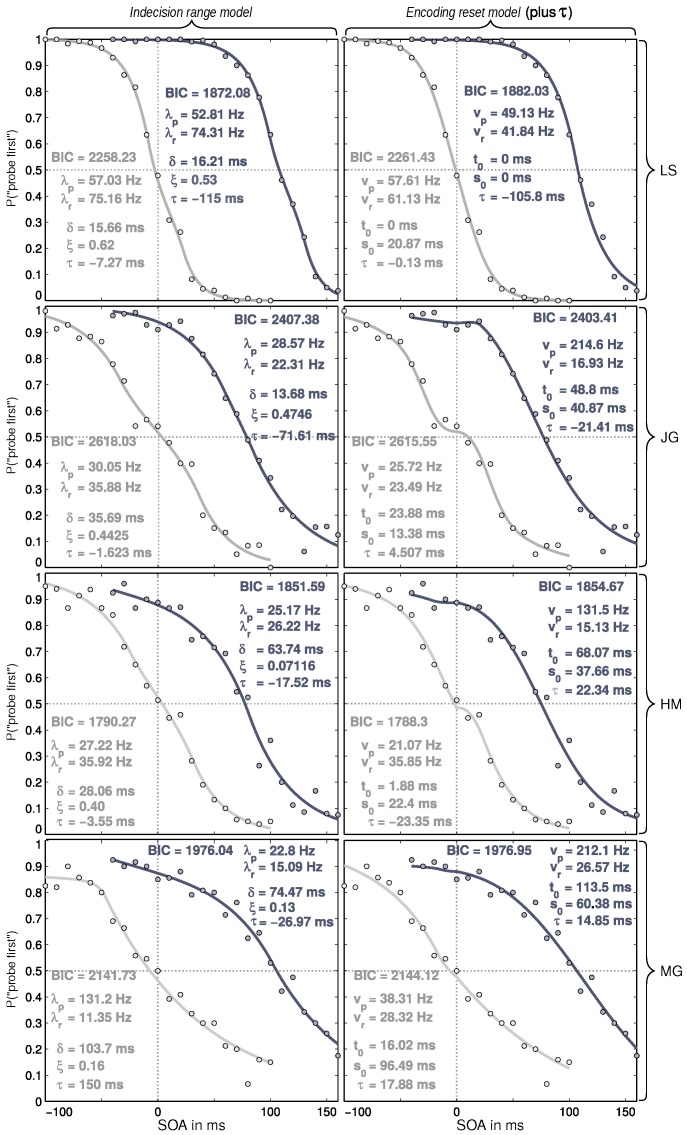
Data, fits, and parameters in Experiment 2. The rows show the different participants, labeled with initials. The columns are the different models, identified in the heading at the top. The cued condition is shown in dark gray, the uncued control condition in light gray. Data points are the frequencies of the judgment “probe first” for the 21 stimulus onset asynchronies (SOAs) studied. In each panel, the respective Bayesian information criterion (BIC) score and all used parameters are given; model parameters as in Figure 3. In addition, parameter τ indicates the position of the plateau on the *x*-axis. For detailed definitions of the parameters, please refer to Section 2.

The estimated parameters and fits are shown in Figure 4. Compared to Experiment 1, there is—except for LS—more variability in the data, especially at SOAs at which the data converge against 0 and 1. The fits are good (note that 21 data points are fitted with five parameters each).

As expected, attention towards one of the targets induced prior entry. This is visible in the clear shift of the cued psychometric distribution towards the right (see Figure 4) and captured by the model fits. One might thus expect that the good fit of the models would also yield reasonable parameter values. The picture is, however, rather complex; we will describe and discuss it in detail in the next paragraphs.

As above, we will first discuss the parameters. Before turning to their estimates, the accuracy of the data for the parameters estimation must be addressed. Compared to the participants of Experiment 1, those of the present experiment are considerably less well trained. Moreover, there were substantially fewer repetitions of each SOA. This is because the overall amount of trials had to be divided between two conditions (attention and neutral) and because, except for LS, participants were available only for five or seven sessions (LS appeared for 10). Overall, this resulted in increased data variability, and, as Figure 4 shows, a considerably worse fit.

The rate estimates show large variability. For the *indecision range model*, the values are between 15 and 131 Hz. Again, these are plausible values, roughly in the range of the first experiment and earlier studies using this method [25]. The rather high value of 131 ms is from participant MG who made lot of errors (visible in the shallow slope and the axes of convergence). This cannot be an appropriate estimate—it stems from the neutral condition where no systematic speed difference is possible, and thus is possibly to the variability in the data. For participant LS, most of the cue-induced shift of the psychometric function is captured by a large τ value, whereas the rates show a 25 Hz advantage of the uncued stimulus. The other three subjects show more moderate τ values in the cued condition and consume more of the shift by a relative processing speed advantage of the probe (measured in λs). The different patterns in the λ and τ parameters may be due to interactions between them. That a relatively large variability can result from this can be seen in the occurrence that in the neutral condition relatively large τ values (which should be at zero) and relatively strong rate differences are found (the rates should be equal). You may also note that, overall, λ estimates are smaller in the cued than the uncued condition.

The *encoding reset model* deals well with the control condition, but not with the data from the cued condition. In the uncued condition, its values are comparable to those derived from the *indecision range model*. In the cued condition, it produces interpretable speed estimates only for participant LS. For the other three, it results in extremely high probe processing rates. A probe which is processed faster than the reference is the expected pattern because the cue guides attention towards the probe. However, the values are absurdly large. The overall processing capacity ($v_{p}+v_{r}$) is up to four times as large as in the neutral condition. With these targets and cue, the overall capacity is not expected to change substantially (cf. [16]). The model does not seem appropriate to capture what is happening in the cued condition.

The τ parameter captures prolongations or reduction in processing latencies of the cue compared to the uncued stimulus. It should be zero in the uncued condition in which no such advantage or disadvantage can arise from a systematic influence. For both models, τ assumes rather small values. However, they are not as close to zero as in the previous experiment, and, in one case, absurdly large: 150 ms (for the *indecision range model*; again participant MG). These deviations from the theoretical value are further indications of the influence of having fewer repetitions than in Experiment 1.

The amount of prior entry caused by a peripheral cue is subject to individual variation, which is also the case in the present experiment. The *indecision range model* gives τ values of −18 to −115 in the cued condition ms, which are all plausible. Note, however, that visibly similar data (compare JG and HM) lead to very different τ estimates. The *encoding reset model* deals better with the τ parameter in the attention condition in these participants. However, it yields a positive τ for JG, which is, given the overall data pattern, very strange.

Again, as the other parameters differ in meaning between the two models, they will be discussed without comparison.

The δ parameter in the *indecision range model* directly corresponds to the range of indecision. Its values range between 14 and 104 ms. Approximately half of the values are above 30 ms, which is rather implausible, looking at the estimates from synchrony judgments discussed above. The vertical plateau location varies largely including values of 0.13 and even 0.07, which indicate a very strong response bias for the cued target. This means that the participants’ ranges of indecision are located at SOAs different from zero (that is expected if prior entry arises) and the bias results in a vertical rise of the plateau.

The $t_{0}$ values of the *encoding reset model* range from 0 to 113 ms with only few values being plausible (a few ten milliseconds), most others being implausibly large and some too small. The variance parameter $s_{0}$ yields highly variable values of 0 to 60 ms.

To give a broad explanation, both models appear to capture the cue-induced prior entry by producing a broad plateau. In the *indecision range model*, this also includes a strong response bias in favor of the attended target (ϵ close to 0). In terms of parameters, however, the results are implausible, especially for the *encoding reset model*.

Comparing the BIC scores for the neutral condition provides a mixed picture. Participants LS and MG are approximated better with the *indecision range model*, participants JG and HM show better fits with the *encoding reset model*. For the attention conditions, the *indecision range model* is preferred for three participants, but not for JG. What is much more important than model fit are, however, the substantive interpretations of the parameters. Here, the *encoding reset model* does not fare well, even though its stimulus encoding model, without the reset, has been successfully used to understand processing in TOJs with attention manipulation by peripheral cues or local contrasts in several papers [15,16,17,18,19,20,21].

The results demonstrate that model comparison presupposes extremely precise data. Only for participant LS do the fits yield reliable estimates. For the other participants, it seems as if the fits respond to the variability in the data with interactions between the parameters, which are neither consistent nor interpretable. Besides collecting more data per participant, using Bayesian statistics with priors informed by earlier studies might provide a remedy.

## 4. Discussion

The two experiments described above investigated distortions in psychometric distributions that are usually attributed to deterministic decision rules. Following up upon the study of Neumann and Scharlau [5], we demonstrated that there is a central, albeit often very narrow, plateau in purely visual TOJs. Using very precise data, the first experiment showed that a processing reset elicited when the second stimulus follows a first one within a short interval seems to be a reasonable explanation. This interval is captured by the parameter $t_{0}$ in the TVA-based TOJ model: if the second stimulus appears within the $t_{0}$-interval of the first stimulus, its processing is reset and both stimuli start again with a common onset. According to TVA, this is because the $t_{0}$-interval supplies time for calculations of the resources, which are later provided to the stimuli. The *indecision range* model can also explain the distortion. It puts it down to an entirely different cause, a range of indecision in which observers are unable to discriminate temporal order and have to guess.

The second experiment tested the competing models in a situation where attention is not equally distributed, but biased towards one target. Above all, the experiment showed that it is impossible to decide between the models on the basis of a reasonably sized data set. Although we strongly recommend using and comparing models in psychological research, such limits should be taken into account before deciding upon methods. Secondly, each of the models produced strange results for at least some of the participants. These problems are possibly due to interaction between each model’s parameters, as can be seen, for instance, in the implausibly small speed parameters for the probe compared to impossibly large ones of the reference in the *encoding reset model’s* results in Experiment 2. As mentioned above, parameters which improve model fit can be easily added to models. However, it is important not to compromise the advantages of a modeling approach by introducing parameters which improve the fit without being theoretically justified. We may have inadvertently done this concerning the τ parameter in the *encoding reset model*. It was added as a simplification of the more complex mechanisms that lead to psychometric function shifts due to cues (cf. [17]). However, beyond capturing the shift, the parameter appears to interact with the processing speed parameters in a theoretically unjustified manner. In addition to avoiding simplifications in subsequent research, the data precision should be increased in order to clarify whether such interactions are caused by the noise in the data.

In summary, we compared two models concerning plausibility and consistency of their parameter estimates and with a formal model comparison criterion. The result is a mixed picture and far from conclusive. In specific cases, one or the other model led to better results. Concerning these comparisons, and model-based analysis in general, an important and often overlooked point must be considered: parameter consistency and formal criteria are not the only—and perhaps not the most important—factor when we consider substantial, theory-based models. For models that merely describe the shape of the data distributions of particular experimental paradigms (e.g., common psychometric functions), goodness of fit and parsimony may be sufficient to decide between competing models. However, when explanative models are derived from theory, as is the case for our TVA-based approach, further factors play a role: does the model fit well with existing theory? Are new (and maybe ad hoc) assumptions indeed required, and can they be used beyond the situation which necessitates their addition? Do estimates agree with those from other paradigms? Does the overall theory explain phenomena beyond a particular effect? These are important issues if the modeling should lead to the accumulation of knowledge and scientific progress. If not taken into account, a special purpose “model” can be tailored for a particular set of data or a class of typical data sets from a particular experimental paradigm, optimizing for goodness of fit and parsimony.

A theory-based, explanative model on the other hand cannot as easily be optimized for particular data and perhaps obscurities within them that are not relevant for the broader picture. Hence, if two models are not on equal levels on the spectrum from deep theoretical integration to superficial reproduction of the current data pattern, it becomes difficult to compare them. In the present study, we have such situation at least to some degree. The *indecision range model* is far from a merely superficial description of the data because it makes various theoretical assumptions, which also have been tested in other domains. However, compared to the TVA-supported approach, it lacks the latter’s deep foundation and mathematical connection to a general theory of attention and (visual) processing, applied across experimental paradigms and clinical research. However, the plateau is a consequence of its original parameters. For the *encoding reset model*, the plateau also originates from a fundamental parameter of the model (TVA’s $t_{0}$), but, it requires that the additional assumption that the reset occurs, a mechanism that has not been experimentally tested elsewhere—at least so far.

In short, a binary “one model outperforms the other” conclusion is difficult in general and not appropriate in the current situation. Both models must be further tested, optimally in new and different settings. That both models considered in this study are similarly successful and have similar drawbacks (especially in the presence of a peripheral cue and noise in the data) may be unsatisfactory now, but it will be a puzzle piece for the broader picture to emerge.

## 5. Conclusions

In summary, while remaining inconclusive on the question of whether the plateau in psychometric functions is generated by a pre-race reset or a post-race decision mechanism, our analysis draws attention to general questions of modeling in cognitive psychology. For future research, the following points should be taken into consideration: (1) For model-based analysis of theoretically important, though subtle differences, very precise data of individual participants is needed, as the data collected in Experiment 1. With attention manipulations, even more repetitions are necessary, rendering data collection extraordinarily expensive; (2) With attention manipulation by peripheral cues, explicit models should be used. The simplification represented by the additional delay parameter τ interferes with the parameters controlling the plateau and should, therefore, be avoided (see Tünnermann and Scharlau [17] for a further description of modeling cued TOJs); (3) It may be advantageous to work with other attention manipulations first. For example, a salience manipulation as in [19] would remove much of the complexity added by the peripheral cue. This could allow the assessment of plateau-producing mechanisms free of the interference from the cue. However, either of the alternatives, explicitly modeling the cue or removing it from the presentation, needs precise data.

On a final note, it may be advantageous to include prior information and conduct a fully Bayesian model comparison. This could help to prevent the models from assuming highly improbable parameter values as they did for the majority of the fits in Experiment 2.

On an even more final note, even though the results (especially from Experiment 2) are far from conclusive—in a sense we are indeed stuck on a plateau—we believe it is important to share our insights in the matter so far. We have tested two models; making the data available with this article, we invite modelers to test their hypotheses on what may generate plateaus in unimodal visual TOJs (see supplementary files for an archive with the data and evaluation scripts). At the same time, we stay on the task of recording a strong TOJ dataset. We plan to improve on the issues of the second experiment by increasing within-subject power, by replacing the interference-prone peripheral cue with a more subtle attention manipulation, and by conducting a hierarchical Bayesian parameter estimation.

### Ethics Statement

Before conducting the experiments, participants read and signed an informed consent. All data were de-identified and analyzed anonymously. This procedure is in line with the ethical guidelines of the “Deutsche Gesellschaft für Psychologie” (German Psychological Society), which states about ethical approval: “In general a request approval of a psychological project is addressed to ethical board if a research funding body (i.e. DFG, VW-Foundation, FP7 of the EU, federal ministries or federal state ministries, foundations, universities) requests an ethical approval for the project. Such a request is usually to be expected if human participants are put at risks or for studies in which human participants are not fully aware about the aims and procedures of the study”. These conditions do not apply to the present study. The experiment was conducted respecting the ethical standards for research with human participants of the *American Psychological Association*. 

## Supplementary Materials

Supplementary materials can be found at http://www.mdpi.com/2411-5150/2/3/29/s1.

## Abbreviations

The following abbreviations are used in this manuscript:

BIC	Bayes information criterion
cm	centimeter
Hz	Hertz
ms	milliseconds
SOA	Stimulus onset asynchrony
TOJ	Temporal-order judgment
TVA	Theory of visual attention
